# Differentiating PNES from Epilepsy- a case report

**DOI:** 10.1192/j.eurpsy.2025.1031

**Published:** 2025-08-26

**Authors:** I. Rakos, D. Lucijanic

**Affiliations:** 1 University Hospital Dubrava, Zagreb, Croatia

## Abstract

**Introduction:**

PNEA- Psychogenic Non-Epileptic seizures resemble epileptic seizures, have no electrophysiological correlate or clinical evidence for epilepsy, whereas there is positive evidence for psychogenic factors that may have caused the seizure. The clinical presentation usually includes convulsive movements, tremor of the whole body, or just some of the parts, loss of awareness, unresponsiveness and sometimes amnesia. ( N.M.G. Bodde, J.L. Brooks, G.A. Baker, P.A.J.M. Boon, J.G.M. Hendriksen, O.G. Mulder, A.P. Aldenkamp, Psychogenic non-epileptic seizures—Definition, etiology, treatment and prognostic issues: A critical review, Seizure, Volume 18, Issue 8, 2009, Pages 543-553, ISSN 1059-1311, https://doi.org/10.1016/j.seizure.2009.06.006.). We were called for a psychiatric consultation for a 35 year old female patient who presented to the Emergency service of our hospital with a history of severe headache unresponsive to painkillers, seizures and a panic attack. A few days prior to this visit she was hospitalized in the Department of Neurology of a different hospital under suspicion of hydrocephalus.

**Objectives:**

The objective of our psychiatric consult was to determine whether the clinical presentation of seizures and headache could be caused by underlying psychological disturbances, rather than by somatic symptoms.

**Methods:**

We reviewed the patient history and previous medical findings and treatment. Additionally, the patient underwent a series of diagnostic tests, with the most important one being video EEG monitoring.

**Results:**

On the MINI diagnostic questionnaire, she met the criteria for Mixed anxiety and depressive disorder and Dissociative and conversion disorder. Psychological testing confirmed a tendency to somatization, and development of secondary psychiatric symptoms on top of the existing physical symptoms. Also, continuous EEG recording for the duration of four days detected only functional seizures, and with other non-pathological findings, among others, through MR of the brain and MR angiography, confirmed the working diagnosis of PNEA.

**Conclusions:**

The mutual cooperation between neurology specialists and liaison psychiatrists is vital in cases like this when there is an unclear cause of the symptoms. Accurate determination of the underlying cause of disturbances enables adequate treatment of the patient.

**Disclosure of Interest:**

None Declared

